# Event and Comment

**Published:** 1913-04-15

**Authors:** 


### DENTAL REGISTER.

**Vol. LXVII. April 15, 1913. No. 4**

### EVENT AND COMMENT.

DELAYED BY FLOODS. This issue of the *Register* is considerably delayed by the great Ohio River flood which put our printing office out of business for ten days.

PROPER PROXIMAL CONTACTS. We are printing in this issue two papers of timely importance on the value of making a proper contact point in fillings, and another on humane methods of separatings for full contour restorations. It may seem like too much of the same subject for our readers, but the timeliness of the subject and the coincident appearance of these valuable papers from the pens of such authorities is our excuse for their presentation. We are apt to feel that the acme of attainment in operating technique has been reached, and that our filling materials are not capable of greater improvement. But our study of function and our ambition to restore the teeth to the best form for ideal function is leading us to more systematic effort in technical attainments. The work of modern orthodontists has taught us the value of ideal occlusion in prosthetic restorations, and the gold inlay is now demonstrating the practicability of typical tooth form restoration within the compass of the average operator. It is now possible to restore lost tooth form at will with less fatigue to both patient and operator and with a more reliable material than was formerly possible, except at great cost of time and with a very high order of skill. The gold inlay is fast supplanting the plastic fillings not only because of better conservative values, but from better esthetic and functional considerations

The introduction of the gold inlay has not, as was at first predicted, in any way lessened the cost of dental operations, for the reason that we have discovered that the construction of gold inlays requires a high order of technical attainment, and that patient and painstaking effort are essential to adequate results. The operation is not capable of being cheapened. In fact there is everywhere a striving to make it better in spite of the fact that this means higher cost. The cheaply made inlay is a disappointment and incompetent operators are going back to the plastics; but the casting process has come to stay, and there will be a strong place for the inlay always in the best practices. The restoration of typical contours, normal occlusion and ideal proximal contacts will be the chief glories of the gold inlay. We therefore trust our readers will read and digest the three papers by Drs. Johnson, Conzett and Ottolengui which we print in this issue. We know of nothing better or more timely for careful consideration at this time.

"CROWN AND BRIDGE WORK A CURSE TO THE PROFESSION". A prominent dentist of the East recently in the discussion of a paper on crown and bridge work, made the statement we have made the subject of this comment. How can this be, or what did he mean by such a sweeping assertion?

We can only guess by reading the discussion and noting the trend of his remarks, for he does not give us a reason for so radical a statement. He evidently had in mind the fact that so much of the crown and bridge work of to-day is faulty in design and technical construction, and ordinarily it would seem that the principles of adaptation for function and sanitation were wholly ignored; consequently the patient is not only inadequately served, but actually, in some cases, injured, esthetically, functionally and hygienically. We can not deny that this is all too true in much of the work one sees; but because this state of affairs obtains to any considerable extent are we justified in condemning a procedure that in capable hands is of such prosthetic value as crown and bridge work has proved itself to be in perhaps the larger majority of cases where it has been used? We have all seen miserable applications of both crown and bridge work which deserve the severest condemnation; but has not the gold filling and every other principle of practice been shamefully misused and abused throughout all our history. Amalgam, vulcanite, cocaine, arsenic and scores of other most useful methods and materials have suffered at the hands of the tyro or quack. Are we ready to condemn these, or any helpful procedure that will add to our resources, simply because some will not take the trouble to master their technique? Bridge work is one of our oldest prosthetic methods and it will survive in spite of the lazy and ignorant, because its principle is sound and its practice will become correct.

